# A Case of Cocaine Toxicity From Body Stuffing

**DOI:** 10.7759/cureus.47805

**Published:** 2023-10-27

**Authors:** Justin Smith, James Espinosa, Alan Lucerna, James Lee, Henry Schuitema

**Affiliations:** 1 Emergency Medicine, AtlantiCare Regional Medical Center, Atlantic City, USA; 2 Emergency Medicine, Jefferson Health, Stratford, USA

**Keywords:** ed management cocaine bodystuffing, cocaine overdose, cocaine bodystuffer management, cocaine toxicity, body stuffing of cocaine

## Abstract

Here, we report a case of body stuffing leading to severe cocaine toxicity. Medical management and supportive care are usually the best course of action in cases of body stuffing, as seen in our case. While surgery is rarely indicated, surgical consultation should occur early to ensure prompt intervention if obstruction or perforation occurs.

## Introduction

Body stuffing refers to the ingestion of an illegal substance in an effort to conceal it and avoid prosecution. This is distinct from, and more common than, the practice of body packing, in which large amounts of an illegal substance are carefully packaged and ingested to be transported discreetly by smugglers [1]. While body packers tend to ingest much larger quantities of a drug, they are less prone to developing symptoms of drug toxicity due to the meticulous packaging methods used when compared with those who ingest packets of drugs in haste to avoid prosecution [2]. Many substances have been reported to be contained in these ingested packets, but cocaine continues to be the most common [1]. While many patients who present to the emergency department (ED) with suspected cocaine body stuffing will be asymptomatic, it is clear that the consequences can have significant medical consequences. About 30% will develop symptoms of sympathomimetic drug intoxication, and 4% will develop severe symptoms, including seizures [3]. Although data exists concerning the epidemiology of cocaine overdoses in the United States, the epidemiology of cocaine body stuffing is unclear. Most of the data appears to be from case reports. It is clear that the consequences can have significant medical consequences. Here, we report a case of body stuffing leading to severe cocaine toxicity. This case was presented in poster format at the Rowan Research Day on May 6, 2021.

## Case presentation

A 22-year-old male presented to the ED with police and emergency medical services (EMS) for altered mental status. Police reported that the patient began acting strangely shortly after being placed under arrest. Prior to arrival in the ED, the patient had admitted to swallowing approximately 40 bags of crack cocaine in the back of the police car. EMS reported the patient had vomited en route to the ED, and they had noticed approximately 15 plastic bags filled with a white substance in his vomitus.

On arrival at the ED, the patient was confused and only intermittently responding to questions. He was actively retching and drooling. He was unable to provide any further history, and a review of his medical record revealed no previous visits or documented medical history. His initial vital signs were a blood pressure of 167/121, a heart rate of 163, a respiratory rate of 30 breaths per minute, a temperature of 96.2 °F orally, and an oxygen saturation of 100% on room air.

The decision was made to intubate the patient for airway protection given his altered mental status and apparent inability to tolerate his own secretions. The patient was intubated using midazolam, fentanyl, and rocuronium. No foreign body was visualized in the airway on video-assisted laryngoscopy. An orogastric tube was placed. A midazolam infusion was started, and his blood pressure had improved to 140/85 at the time of admission.

The patient’s urine drug screen was positive for cocaine, phencyclidine, and cannabinoids. His ethanol level was <10 mg/dL. The patient’s potassium was 3.0 mmol/L. This was replaced with 20 mEq of intravenous potassium chloride. His serum glucose was 167 mg/dL. His initial lactate was 3.5 mmol/L. Arterial blood gas after intubation was a pH of 7.19, pCO_2_ of 75 mmHg, pO_2_ of 257 mmHg, and bicarbonate of 29 mmol/L. The labs were otherwise unremarkable. A non-contrast computerized tomography (CT) scan of the chest, abdomen, and pelvis was performed, which showed no acute findings. Critical care, general surgery, and gastroenterology were consulted. The patient was admitted to the intensive care unit (ICU).

Shortly after arriving in the ICU, the patient was awake and agitated but was following commands. He was later able to be extubated. He had another episode of vomiting while in the ICU, and plastic packets were again noted in the vomitus. Midazolam was discontinued following extubation, and the patient remained normotensive. His high-sensitivity troponin rose from 3 ng/dL to 20 ng/dL. He had a CT scan with oral contrast the next morning, which was normal. He had a bowel movement later that day that appeared to have pieces of plastic within it. He was downgraded from the ICU on hospital day 2 and discharged from the hospital on hospital day 3.

## Discussion

Body packers will typically present to the emergency department for one of three reasons: symptoms of a drug toxidrome, symptoms of bowel obstruction or perforation, or for evaluation prior to incarceration or detainment. The management of body packers is generally well-established. Asymptomatic patients are conservatively managed with laxatives until imaging confirms that all packets have been retrieved. Surgery is performed if the patient develops an obstruction or perforation or if there is any sign of drug toxicity given the enormous amounts of drug in each packet [4,5]. Management and disposition of cocaine body stuffers, on the other hand, pose considerable challenges and are not as well-established [2,6].

Most patients presenting to the ED with suspected body stuffing will be asymptomatic or have a tachycardia that resolves [3,7]. Given the high risk of drug leaking from poorly prepared packets and the lack of an antidote to cocaine overdose, most experts recommend treating these patients with 1 g/kg of activated charcoal. While cocaine is rapidly absorbed through the intestinal mucosa, activated charcoal has been shown to reduce the incidence of seizures and death in mouse models [8]. There is some evidence to support discharge after a six-hour observation period in patients who have received activated charcoal, but there have been case reports of patients developing severe symptoms 24 hours after ingestion [2,7,9]. In addition to activated charcoal, whole-bowel irrigation with 1.5-2 L/hour of polyethylene glycol electrolyte solutions (Go-Lytely) should be initiated, and stools should be examined for foreign bodies. Whole-bowel irrigation is contraindicated in patients who have bowel perforation, ileus, or bowel obstruction. It is also contraindicated in patients with hemodynamic instability and compromised, unprotected airways [10]. CT of the abdomen and pelvis can be used to evaluate the number of packets swallowed on presentation or to confirm the expulsion of all packets after whole bowel irrigation [2]. A CT scan, with contrast or non-contrast, can fail to identify packets. Hahn et al. point out that multiple modalities may be needed to increase confidence that a therapeutic endpoint has been reached [11].

In cases of body stuffing, most patients will have begun to exhibit signs of intoxication by the time they present to the emergency department [7]. Patients who develop symptoms of intoxication after body stuffing are better managed medically rather than with surgery. Cocaine is a sympathomimetic drug that acts on adrenergic receptors both centrally and peripherally. Symptoms of cocaine ingestion range from tachycardia and hypertension to agitation, hyperthermia, myocardial infarction, and seizures. Patients with mild to moderate symptoms of intoxication should be treated with benzodiazepines. If hypertension persists despite benzodiazepines, nitroglycerin or nitroprusside may be effective. β-blockers should be avoided as they cause unopposed activation of α-adrenergic receptors and have been shown to increase cardiac mortality [2]. If the patient develops severe cocaine toxicity, such as repeated seizures, hyperthermia, severe agitation, or myocardial ischemia, he or she will require admission to the ICU and continued medical management [2,7].

Surgery is indicated for bowel obstruction, intussusception, or signs of ischemic bowel. The surgical team should nevertheless be consulted early before complications develop [2]. Endoscopy can be used to look for packets that remain in the stomach, but endoscopic removal of these packets risks rupturing the packages and is therefore dangerous [1,2]. Whole-bowel irrigation can be continued until the rectal effluent becomes clear [10].

## Conclusions

Body stuffing with cocaine can have severe medical consequences. Medical management and supportive care are usually the best course of action in cases of body stuffing, as seen in our case. Surgical consultation should occur early to ensure prompt intervention if obstruction or perforation occurs.
